# Association of Preoperative Diagnosis with Clinical Yield of Muscle Biopsy

**DOI:** 10.7759/cureus.3449

**Published:** 2018-10-14

**Authors:** Steven O Tenny, Kyle P Schmidt, Kenneth A Follett

**Affiliations:** 1 Neurosurgery, University of Nebraska Medical Center, Omaha, USA

**Keywords:** muscle biopsy, therapeutic yield, clinical yield, preoperative differential diagnosis

## Abstract

Background: Muscle biopsy is a common diagnostic marker for myopathy assessment; however, it has a relatively low pathologic yield of less than 60%. Additionally, both diagnostic and non-diagnostic muscle biopsies can provide guidance for treatment, i.e, provide therapeutic usefulness.

Purpose: We designed a study to determine if having a documented definitive preoperative differential diagnosis would affect the pathologic yield and therapeutic usefulness of muscle biopsies for myopathy.

Methods: This was a retrospective, single institution chart review of 106 consecutive muscle biopsies in adult patients, which looked at the presence or absence of a definitive preoperative differential diagnosis and relation to diagnostic yield and therapeutic usefulness of muscle biopsies.

Results: Of 106 muscle biopsies, 50 biopsies (47%) had a definitive preoperative differential diagnosis, 52 biopsies (49%) returned definitive pathology, and 93 biopsies (88%) provided therapeutic information. The presence of a documented differential diagnosis increased the odds of pathologic yield by 3.73 (p-value < 0.01) and therapeutic usefulness by 3.40 (p-value 0.08). If pathology was diagnostic then the therapeutic usefulness of the biopsy was 4.54 times more likely (p-value < 0.01).

Conclusion: Documentation of a definitive preoperative differential diagnosis, when pursuing muscle biopsy for myopathy, is associated with an increased pathologic diagnostic yield. Definitive pathology was associated with an increase in the therapeutic usefulness of the muscle biopsy.

## Introduction

Muscle biopsy is a useful tool in the evaluation of neuromuscular conditions, especially myopathy, and is often considered the gold standard for diagnosis [1,2]. However, muscle biopsy is associated with inherent surgical risks and notable cost compared with non-surgical evaluations, including history and physical examination, laboratory evaluation, imaging, nerve conduction study (NCS) or electromyography (EMG) [1,3-6]. If a non-surgical workup for myopathy is inconclusive then a muscle biopsy may be needed [3,7]. Unfortunately, even with an extensive preoperative evaluation muscle biopsy does not always provide a diagnostic biopsy [2-4,7-11].

In order to reduce the procedural risks, while maximizing the utility of muscle biopsy, it is necessary to review all preoperative factors to identify patients who are more likely to have a pathologically diagnostic biopsy or therapeutic impact based on the biopsy. While certain preoperative factors have already been analyzed in the literature [4], one aspect which has not been evaluated, to the best of our knowledge, is the documentation of a definitive differential diagnosis list. We therefore set out to determine if the presence of a documented differential diagnosis increased the therapeutic or diagnostic yield of a muscle biopsy using a retrospective chart review.

## Materials and methods

We performed a retrospective chart review with the inclusion criteria of all patients age 19 and older who had a muscle biopsy performed between January 1, 2012 and July 1, 2016 at a single academic tertiary care hospital. We ran a query for all patients who had Common Procedural Terminology (CPT, American Medical Association (AMA®) 2016) code 20200 (superficial muscle biopsy) or 20205 (deep muscle biopsy) listed as a diagnosis code for surgery.

All biopsies were performed by an academic neurosurgeon. For each muscle biopsy two separate segments of the same muscle were obtained with muscle biopsy clamps and excised sharply and given to pathology for processing. All of the muscle biopsy specimens were examined by board certified neuropathologists.

For all patients identified, the chart was abstracted for age at biopsy, gender, muscle biopsied based on the operative report, preoperative differential diagnosis by the referring service, pathology of the biopsy, and therapeutic implications of the muscle biopsy result.

The preoperative differential diagnosis was dichotomized into either being a definitive differential diagnosis set or a non-definitive differential diagnosis set. A definitive differential diagnosis set consisted of a differential with at least some specific pathologic entities documented as the reason for the muscle biopsy. A non-definitive differential diagnosis set consisted of a differential diagnosis with only non-specific entities listed.

Pathology reports for the muscle biopsy were dichotomized into definitive versus non-definitive pathologic results. Definitive pathologic results were reports which identified a specific disease process. Non-definitive pathology results included either a list of possible diagnoses or listed descriptors of the histology without making a specific diagnosis.

The therapeutic usefulness of the biopsies were likewise dichotomized into either therapeutically useful pathology results or not therapeutically useful results. Notes and treatment plans from the referring service were reviewed for one year after the date of the muscle biopsy pathology report. The pathology was considered therapeutically useful if there was any documentation citing the muscle biopsy either alone or with other supporting evidence to continue, modify, or halt treatments such as steroids, intravenous immunoglobulin, immunosuppressive medications, other medications, or other therapies. A pathology result was deemed not therapeutically useful if it was either not mentioned in any treatment notes within one year or stated to have no impact on clinical treatment decisions.

The categorical variables of definitive preoperative differential, diagnostic pathology and therapeutically useful results were compared using a two-tail Fisher's exact test with a prespecified alpha of 0.05. Statistical analysis was carried out by a statistician using SAS versions 9.4 (SAS Institute Inc., Cary, NC). Prior to any chart searches or data retrieval, the project was submitted to and approved by the institution's Institutional Review Board (IRB 502-16-EX).

## Results

We identified a total of 106 muscle biopsies during the 54-month period of the study. The ages for patients included in the study ranged from 21 to 86 years with a mean of 57.4 years and standard deviation of 16.9 years. The gender of the study group was as follows: 54.7% males and 45.3% females. Of the 106 biopsies, there were 34 vastus lateralis, 27 quadriceps, 18 deltoid, nine biceps, four rectus femoris, three paraspinal, three triceps, three vastus medialis, two gluteal, and one each of gastrocnemius, hamstring, and trapezius muscles biopsied. The referrals for muscle biopsy came from neurology (68%), rheumatology (29%), family medicine (1%), oncology (1%), and gastroenterology (1%).

Referring services had a definitive differential diagnosis in 50 of 106 cases (47%). Pathology was definitive in 52 of 106 biopsies (49%) and therapeutically useful in 96 of 106 cases (88%).

Table 1 shows the definitive pathology stratified by presence or absence of a definitive preoperative differential diagnosis from the referring service. For the 50 cases with definitive preoperative differential diagnosis, 33 cases (66%) had definitive pathology. For the 56 cases where there was not a definitive preoperative differential diagnosis, 19 cases (34%) had definitive pathology for the muscle biopsy. The p-value for the association is 0.0017 with an odds ratio of 3.73 (95% CI 1.676-8.529) for the biopsy showing definitive pathology if a definitive preoperative differential diagnosis was documented versus no definitive differential diagnosis documented. Documentation of a definitive preoperative differential diagnosis provides a sensitivity of 63% and specificity of 69%. Additionally, documentation of a definitive preoperative differential diagnosis provides an absolute risk reduction of non-diagnostic muscle biopsy by 32%, giving a number needed to treat (NNT) of 3.125.

**Table 1:** Definitive Preoperative Differential Diagnosis Versus Definitive Pathology

Definitive Preoperative Differential	Definitive Pathology		
	Yes	No	Total
Yes	33	17	50
No	19	37	56
Total	52	54	106
	p-value		0.0017

Table 2 shows therapeutic usefulness of the biopsy stratified by presence or absence of a definitive preoperative differential diagnosis of the referring service. In the 50 cases where a definitive preoperative differential was present, 47 of the biopsies (94%) were therapeutically useful. This is higher than the 46 (82%) therapeutically useful muscle biopsies of the 56 cases with no definitive differential diagnosis present. The p-value of association is 0.0791 with an odds ratio of 3.369 (95% CI 0.9109 - 16.07) for the biopsy having therapeutically useful information if a definitive differential was documented versus if no definitive differential diagnosis was documented.

**Table 2:** Definitive Preoperative Differential Versus Therapeutic Benefit of Biopsy

Definitive Preoperative Differential	Therapeutic Benefit of Biopsy		
	Yes	No	Total
Yes	47	3	50
No	46	10	56
Total	93	13	106
	p-value		0.0791

Table 3 shows the therapeutic usefulness of the muscle biopsy stratified by definitive pathology being present or absent. All biopsies with definitive pathology were therapeutically useful compared to the 41 therapeutically useful muscle biopsies of 54 total muscle biopsies without definitive pathology (76%). The p-value of association is 0.0001 with an odds ratio of 4.539 (95% CI 4.539 - no limit) for the biopsy having a therapeutically useful result if definitive pathology was identified versus if no definitive pathology was identified. 

**Table 3:** Definitive Pathology Versus Therapeutic Benefit of Biopsy

Definitive Pathology	Therapeutic Benefit of Biopsy		
	Yes	No	Total
Yes	52	0	52
No	41	13	54
Total	93	13	106
	p-value		0.0001

## Discussion

Muscle biopsies are often considered during the workup of myopathy. A muscle biopsy can aid in the diagnosis of numerous pathologic processes including vasculitis, muscular dystrophy, inflammatory myopathy, mitochondrial diseases, and inherited myopathies [1]. Published rates of diagnostic yield for open muscle biopsies in various myopathic conditions ranges from 17% to 60% based on the age group and underlying pathology [2-4,7-11]. With the wide variation in diagnostic yields of muscle biopsies and risk for surgical and anesthetic complications, we believe it is important to identify additional preoperative factors which can improve the diagnostic yield of muscle biopsies.

Our findings show that if a referring service has a definitive differential diagnosis documented then a muscle biopsy is 3.73 times more likely (p-value 0.0017) to show definitive pathology compared to either no documentation of a definitive differential diagnosis or a non-definitive differential diagnosis. Similarly, if definitive pathology is identified on the muscle biopsy then the biopsy is 4.54 times more likely (p-value 0.0001) to have therapeutic usefulness. Previous literature has evaluated various other factors relating to muscle biopsy yield but none examined preoperative differential diagnosis as an important factor. Other factors evaluated in the literature include clinical evaluation with strength testing [2,6], selecting biopsy locations [5,12], biopsy technique used [4,13,14], and simultaneous muscle and nerve biopsies [3,8,15].

We further found that if a definitive preoperative differential diagnosis is present then there is a trend towards increased therapeutic benefit of the biopsy (odds ratio 3.369 and p-value 0.0791). Although this trend did not reach statistical significance, it may warrant further investigation with a larger study.

There may be some underlying reasoning for the associations we observed. For a physician to generate a differential diagnosis, the clinician has to weed through the list of known causes of myopathy or weakness to identify the most likely etiologies for the specific patient. The differential then may be a proxy for adequate preoperative workup prior to referral for biopsy. Alternatively, some physicians may order numerous tests in parallel (labs, imaging, and biopsy) at the same time as opposed to in series (labs then imaging then biopsy), which could result in muscle biopsies being requested and performed prior to completion of the laboratory workup, allowing for narrowing of a differential diagnosis.

With most medical decisions and recommendations based on the differential diagnosis, documentation of such is important prior to pursuing procedural intervention. In several neuromuscular conditions the progression of symptoms may be indolent resulting in a lengthy evaluation with normal or slightly abnormal results making the differential diagnosis initially broad. However, prior to exposing patients to procedural risks the progression of the workup should allow for a definitive differential list to be formulated and documented. Unfortunately, either providers do not always have a definitive differential diagnosis or inadequately document the presence of such when requesting a biopsy, as seen in this study.

Our study has a few limitations. First, we note internal validity is always a concern and this study is no different as all the muscle biopsies were performed by one of five experienced neurosurgeons at an academic institution. All neurosurgeons who performed muscle biopsies in this study had a similar approach and threshold for muscle biopsies. All biopsies were analyzed with consistent means and resources and by board certified neuropathologists.

Two separate reviewers (KPS and SOT) reviewed each preoperative differential, pathologic diagnosis, and therapeutic documentation to determine if each was definitive or not definitive. This helped ensure consistent application of the terms definitive and non-definitive. Any differences of opinion were settled by group consensus.

A second area of concern is external validity. All muscle biopsies were done at a tertiary academic center. Many of the patients had been referred by either an academic neurology or rheumatology department while some of the patients were referred from community physicians. Thus the referral pattern for muscle biopsies may have an effect on the results as more “straightforward” cases of myopathy may have been handled in non-academic settings.

Additionally, a board certified neuropathologist may not be available to interpret muscle biopsy specimens in certain settings. Facilities can also vary in the array of pathologic testing they perform on muscle biopsies, which can change the pathologic yield.

Another possible concern involves prior studies discussing therapeutic yield of muscle biopsies but failing to clarify how therapeutic yield is determined. We have listed our method for identifying therapeutic yield to allow for definitive future comparisons between our study and others.

## Conclusions

If a documented definitive preoperative differential diagnosis is present when working up a myopathic process then obtaining a definitive pathologic yield of a muscle biopsy is 3.73 times more likely than when a differential diagnosis is not documented. Additionally, if there is definitive pathology on a muscle biopsy then it is 4.54 times more likely the treating team will make therapeutically important management decisions. Thus it is important for referring physicians to have a definitive differential diagnosis documented prior to seeking a muscle biopsy in cases of myopathy to increase the pathologic and therapeutic yield of the biopsy.

## References

[1] Hart MG, Santarius T, Trivedi RA (2013). Muscle and nerve biopsy for the neurosurgical trainee. Br J Neurosurg.

[2] Shaibani A, Jabari D, Jabbour M, Arif C, Lee M, Rahbar MH (2015). Diagnostic outcome of muscle biopsy. Muscle Nerve.

[3] Chen DJ, Prayson RA (2014). Evaluation of simultaneous muscle and nerve biopsies for the diagnosis of neuromuscular diseases. Ann Diagn Pathol.

[4] Lai CH, Melli G, Chang YJ, Skolasky RL, Corse AM, Wagner KR, Cornblath DR (2010). Open muscle biopsy in suspected myopathy: diagnositc yield and clinical utility. Eur J Neurol.

[5] Lovitt S, Marden FA, Gundogdu B, Ostrowski ML (2004). MRI in myopathy. Neurol Clin.

[6] Miller S, Shevell M, Silver K, Kramer M (1998). The diagnostic yield of the nerve-muscle skin biopsy in paediatric neurology practice. Pediatr Rehabil.

[7] Reynolds EM, Thompson IM, Nigro MA, Kupsky WJ, Klein MD (1999). Muscle and nerve biopsy in the evaluation of neuromuscular disorders: the surgeon's perspective. J of Pediatr Surg.

[8] Claussen GC, Thomas TD, Goyne C, Vazquez LG, Oh SJ (2000). Diagnostic value of nerve and muscle biopsy in suspected vasculitis cases. J Clin Neuromusc Dis.

[9] Gibreel WO, Selcen D, Zeidan MM, Ishitani MB, Moir CR, Zarroug AE (2014). Safety and yield of muscle biopsy in pediatric patients in the modern era. J Pediatr Surg.

[10] Jamshidi R, Harrison MR, Lee H, Nobuhara KK, Farmer DL (2008). Indications for pediatric muscle biopsy determines usefulness. J Pediatr Surg.

[11] Skram MK, Gulati S, Larsson E, Lindal S, Torp SH (2009). Muscle biopsies in children – an evaluation of histopathology and clinical value during a 5-year period. Ups J Med Sci.

[12] Prayson RA (2006). Diagnostic yield associated with multiple simultaneous skeletal muscle biopsies. Am J Clin Pathol.

[13] Magistris MR, Kohler A, Pizzolato G, Morris MA, Baroffio A, Bernheim L, Bader CR (1998). Needle muscle biopsy in the investigation of neuromuscular disorders. Muscle Nerve.

[14] Campellone JV, Lacomis D, Giuliani MJ, Oddis CV (1997). Percutaneous needle muscle biopsy in the evaluation of patients with suspected inflammatory myopathy. Arthritis Rheum.

[15] Bennett DL, Groves M, Blake J (2008). The use of nerve and muscle biopsy in the diagnosis of vasculitis: a 5 year retrospective study. J Neurol Neurosurg Psychiatry.

